# Editorial

**DOI:** 10.5152/TJAR.2022.010622

**Published:** 2022-04-01

Dear Colleagues,

The Coronavirus 2019 (Covid-19) pandemic has changed our personal and professional worlds in unimaginable and unprecedented ways, with dramatic consequences worldwide in the economic, financial, social, health, and scientific fields. Anaesthesiology, reanimation and intensive care specialists have become frontline specialists in an unusual infectious disease environment, operating in the operating room, intensive care unit, emergency response, and team leaders. 

We have not been accustomed to our new profession, which involves excellent clinical uncertainty and potential personal hazards. Although the chaotic environment has calmed down a bit, we still do not know what will happen in the future, and we think that we should be prepared for anything at any moment.

Published articles and case reports allowed us to increase our understanding of the pandemic and provide better treatment to our patients. To not delay the publication of the papers accepted by our journal about Covid-19 and to contribute to the scientific world, it was suggested to the TARD board of directors to issue an additional issue by the editors of the TJAR journal. This supplementary issue contains 10 original articles, 2 case reports and 3 letters to the editor about the Covid-19 pandemic. 

Despite the uncertainties created by the Covid-19 pandemic, we will inevitably walk in the light of science.

Respectfully yours,

Prof. Dr. Necati Gökmen

On behalf of the editorial board

